# Vermicompost Amendments Disrupt Feeding Behavior of *Diaphorina citri* Kuwayama and Boost Activities of Salicylic Acid and Jasmonic Acid Pathway-Related Enzymes in Citrus

**DOI:** 10.3390/insects14050410

**Published:** 2023-04-25

**Authors:** Tonglai Tao, Zhaohong Wang, Runqian Mao, Mubasher Hussain, Steven P. Arthurs, Fengxian Ye, Xincheng An, Jing Gao

**Affiliations:** 1 School of Life Sciences, South China Normal University, Guangzhou 510631, China; 2 Guangdong Key Laboratory of Animal Conservation and Resource Utilization, Guangdong Public Laboratory of Wild Animal Conservation and Utilization, Guangdong Engineering Research Center for Mineral Oil Pesticides, Institute of Zoology, Guangdong Academy of Sciences, Guangzhou 510260, China; 3 Biobee USA, Altamonte Springs, FL 32714, USA

**Keywords:** vermicompost, Asian citrus psyllid, electrical penetration graph, plant resistance

## Abstract

**Simple Summary:**

*Diaphorina citri* Kuwayama is a phloem-feeding insect that uses piercing–sucking mouthparts to ingest phloem sap from plants. It is the most common citrus pest because it spreads the bacteria that causes huanglongbing during the probing and feeding process. Nowadays, eco-friendly methods such as soil amendment with vermicompost are gaining popularity in the integrated management of *D. citri*. Here, we investigated why vermicompost amendments cause negative effects to *D. citri*. We observed that vermicompost disrupted *D. citri* feeding behavior, particularly for the variables related to phloem sap ingestion, with a significant reduction in the duration of phloem sap ingestion as well as a longer time spent to reach and gain access to phloem sap. Additionally, we demonstrated that vermicompost amendments increased plant defense-associated enzyme activities. We speculate that vermicompost amendments enhance plant resistance against *D. citri*, which disrupts the insect feeding and eventually impairs the performance of *D. citri*. Furthermore, we showed how vermicompost amendments affect the feeding behavior of piercing–sucking insects for the first time, and the feeding behavior (decreased efficiency of phloem sap ingestion) suggests vermicompost may suppress the transmission of huanglongbing bacteria by *D. citri*, yet it still needs to be further studied.

**Abstract:**

Plants grown with vermicompost amendments are known to be harmful to sap-sucking insects, but the underlying mechanism remains to be determined. Here we investigated the feeding behavior of *Diaphorina citri* Kuwayama on *Citrus limon* (L.) Burm. F using the electrical penetration graph technique. Plants were grown in soil with different vermicompost rates (0%, 20%, 40%, and 60% *w*/*w*). Additionally, plants were tested for the activity of salicylic acid (SA) and jasmonic acid (JA) pathway-related enzymes. When compared to the control, vermicompost treatments (40% and 60%) decreased duration of phloem sap feeding and increased duration of the pathway phase of *D. citri*, and the 60% vermicompost made it more difficult for *D. citri* to reach and gain access to phloem sap. Enzymatic assays indicated that the 40% amendment rate increased phenylalanine ammonia lyase (involved in the SA pathway) and polyphenol oxidase (involved in the JA pathway), while the 60% amendment rate increased -1,3-glucanases (involved in the SA pathway) and lipoxygenase (involved in the JA pathway). The 20% amendment rate had no effect on feeding or enzyme activities. This study revealed that vermicompost amendments can reduce the efficiency of *D. citri* feeding, which may result from increased plant resistance via the SA and JA pathway.

## 1. Introduction

Vermicompost is a fertilizer produced from organic wastes through the joint action of earthworms and microorganisms [1]. The application of vermicompost can improve soil porosity and stability, restore nutrients, and promote plant growth and yield [2,3,4]. In addition, vermicompost applications can suppress the performance and damage of herbivores, particularly among sap and phloem-feeding arthropods. For example, potting media amended with vermicompost suppressed population of the two-spotted spider mite *Tetranychus urticae*, mealy bug *Pseudococcus sp*., and green peach aphid *Myzus persicae* [5]. The development and fecundity of melon aphid *Aphis gossypii* [6] and the whitefly *Bemisia tabaci* [7] were impaired by vermicompost treatments. 

Although the suppressive effects of vermicompost against herbivorous insects is well documented, few studies have investigated potential mechanisms. Yatoo (2021) suggests that the vermicompost composition, such as nutrients, beneficial microbes, entomopathogens, and predatory nematodes, functions in suppressing the incidence of herbivores [2]. Some beneficial ingredients from vermicompost may interact with plants and induce the production of defensive compounds, which may enhance plant resistance to herbivorous insects. For example, the increased phenol contents in Bell Pepper *Capsicum annuum* amended with vermicompost were associated with the decreased life table parameters of *M. persicae* [8]. Increased phenols and defensive enzymes (viz. polyphenol oxidase and peroxidase) were also suggested to be responsible for the decreased incidence of leafhopper *Amrasca biguttula biguttula* and whitefly *B. tabaci* on sunflower, *Helianthus annuus*, under vermicompost amendments [9]. In addition, the negative effects of vermicompost on the performance of herbivores may arise from the inhibited feeding of herbivorous insects. Fong (2022) reported that vermicompost inhibited the feeding and food conversion efficiency of tobacco cutworm *Spodoptera litura* (a chewing insect) on *Brassica chinensis* [10]. How vermicompost affects the feeding of piercing–sucking insects is unknow. 

The piercing–sucking insects such as aphids and psyllids use the long-mouthpart to penetrate cells and ingest phloem sieve elements. However, the plant is not always passively being fed. Actually, plants have developed sophisticated defenses against insects, which are mainly regulated by the phytohormonal pathways such as salicylic acid (SA) and jasmonic acid (JA) [11]. These phytohormone-dependent defenses can inhibit the feeding of insects [12,13]. Systemic resistance against insect herbivores also can be induced by other factors, such as the soil amendment of silicon [14] and microbes [15,16]. As vermicompost can contain a diverse array of macro/micro-nutrients and microbes [1], it is conceivable that it may also influence phytohormone-dependent defenses and the feeding behavior of piercing–sucking insect herbivores. However, it remains unexplored. 

The Asian citrus psyllid (*Diaphorina citri* Kuwayama) is currently the most serious insect pest for citrus. The primary notoriety of *D. citri is* due to its transmission of the phloem-limited bacteria *Candidatus Liberibacter* asiaticus (*C*Las), which is associated with huanglongbing (HLB) [17]. The feeding behavior of many piercing–sucking hemipteran plant pests (such as the aphid, whitefly, and psyllid) can be monitored in real-time using the electrical penetration graph (EPG) technique [18]. This technique helps in elucidating how abiotic factors (such as insecticides, plant defense inducers, and climate change) affect the performance of insect pests and the acquisition/inoculation of insect-vectored pathogens [19,20,21]. 

When feeding on citrus treated with vermicompost, the *D. citri* population was reduced just as that of the majority of insects [22,23]. We speculate that this may be caused by modifications in *D. citri*’s feeding behavior. In this study, we investigated the feeding behavior of *D. citri* that feeding on citrus grown in soil amended with various levels of vermicompost (0, 20%, 40%, and 60%). We also studied how applying vermicompost affected the activities of enzymes involved in the SA and JA pathways in citrus infested and uninfested with *D. citri*.

## 2. Materials and Methods

### 2.1. Vermicompost and Soil

Vermicompost was purchased from Guangdong Zhongshi Longtai Low Carbon Technology Co., Ltd., Guangzhou, China. The field soil (0–30 cm depth) was collected from a citrus orchard in the Conghua district of Guangzhou city. The soil was blended before using. Vermicompost was applied to field soil and tested on plants at the rates of 0% (control), 20%, 40%, and 60% (*w*/*w*, humidity: 55% and 70% for vermicompost and soil respectively). 

### 2.2. Plants and Insects

*Citrus limon* (L.) Burm. F. seedings (20 cm in height) were purchased from a nursery in Beihai, China. The seedlings were individually planted in 5 L plastic pots, containing vermicompost amended soils. Plants were maintained in a climate-controlled room at 25 °C, 16 h:8 h (light: night) and 60–70% humidity. These plants were watered twice a week and were used for experiments two months later. 

The *D. citri* used in this study were collected and maintained on *Murraya paniculata* (L.) Jack from Sun Yat-sen University at Guangzhou, China. The *M. paniculata* and *D. citri* were placed in a separate climate-controlled room, with the same condition as described above. The *C*Las free status of *D. citri* was confirmed using a qPCR-based method [24]. 

### 2.3. Feeding Behavior of D. citri

The feeding behavior of adult female *D. citri* was recorded using a 4-channel direct-current EPG system (Giga-4; EPG systems, Wageningen University, Wageningen, The Netherlands) [13]. The analog EPG signal was converted to digital through a A/D converter (DI-710, Data Instruments Inc., Akron, OH, USA). Data acquisition and analysis were performed with stylet+ software (version 3.1.9) (Wageningen University, The Netherlands).

Female *D. citri* were selected for use. A single *D. citri* was tethered to a gold wire (2 cm long and 12.5 µm diameter) of the equipment at the pronotum with conductive silver glue. Then the *D. citri* was placed on the abaxial side of the fully expanded young leaf (9 cm in length and 4 cm in width) of the plant. To complete the circuit, another electrode (plant electrode) was inserted into the soil. All the experimental set-up was placed in a Faraday cage, and the feeding behavior was recorded for 8 h. One adult and one plant constituted a test, and 12 tests (replicates) were conducted for each treatment. 

To quantify feeding behaviors, six distinct waveforms were classified for *D. citri*: non-probing (Np); pooled pathway phase activities (C, including epidermis first stylet contact, intercellular sheath salivation, and stylet movements), first contact with phloem (D), salivary secretion into sieve elements (E1), phloem sap ingestion (E2), and xylem sap ingestion (G) [25]. Response variables were calculated according to that reported in aphids and psyllids, include the number of waveform events per insect (NWEI) and the mean waveform duration per insect (WDI) [26]. In addition, other variables associated with plant resistance to insects were calculated [27]: the time to first probe from the start of recording (reflecting surface resistance), the minimum duration of C before first E1 (related to epidermis/mesophyll resistance), the time to first E1 and time to first E2 (reflect the mesophyll/phloem resistance), and the average duration of E2 (reflect phloem resistance).

### 2.4. Activity of Key Enzymes in the SA and JA Defense Pathway

Eight plants were assigned to each of the four vermicompost treatment. Four plants received *D. citri* infestation and four plants remained uninfested. Plants with expanded young leaf (about 4 cm wide and 9 cm long) were infested with 0 (uninfested control) and 50 adult female *D. citri* (only one leaf from one plant was used). Leaves were covered with 80-mesh nylon net (9 cm wide and 12 cm long). After 12 h, the exposed leaves were excised, placed in liquid nitrogen, and then stored at −80 °C for enzyme activity analysis. 

The phenylalanine ammonia lyase (PAL) and β-1,3-glucanases (involved in SA pathway), as well as lipoxygenase (LOX) and polyphenol oxidase (PPO) (involved in JA pathway) were investigated. Enzyme activities were determined using a corresponding assay kit (Sangon Biological Engineering Technology and Services Co., Ltd., Shanghai, China). Tissue samples (0.05 g) were grounded in a 0.5 mL extraction solution, and the mixture was centrifuged at a low temperature to obtain the crude enzyme extract. The supernatant was used to determine the enzyme activity with a spectrophotometer according to the manufacturer’s procedure of each kit. 

### 2.5. Statistical Analysis

Statistical analysis was performed using SPSS 20 software (SPSS Inc., Chicago, IL, USA). Two-way analysis of variance (ANOVA) was used to analyze the effect of the vermicompost rate by *D. citri* infestation on the defense enzyme activities. One-way ANOVA (followed by Tukey post-hoc) was used to analyze the effect of vermicompost on the EPG variable values and defense enzyme activities. Before ANOVA analysis, data were square-root or log transformed to meet assumption of normality and homogeneity of variances. The student’s *t*-test was used to analyze the difference of each defense enzyme activity in leaves with and without *D. citri* infestation. Results were considered significantly different at *p* < 0.05.

## 3. Results

### 3.1. Feeding Behavior of D. citri

For the number of waveform events per insect (NWEI), only sustained phloem ingestion (E2 > 10 min) was significantly different among treatments (Table 1). The sustained E2 event was reduced by 50% at the 40% amendment rate and 55% at the 60% amendment rate compared with the control, while it was not significantly affected by the 20% amendment rate. There were no significant differences in the number of Np, C, D, E1, E2, or G among the treatments.

The EPG data showed that the vermicompost treatments affected the mean waveform duration per insect (WDI) of the several waveforms (including C, E2, and sustained E2) (Table 2). Specifically, for the 40% and 60% amendment rates, the pooled route phases (waveform C) were increased by 37% and 44%, respectively, compared to the control. At the corresponding 40% and 60% amendment rates, phloem intake (E2) was decreased by 38% and 44%, respectively. The sustained E2 was reduced by 83% at the 60% amendment rate. The xylem phase (G) waveform was only observed by two individuals feeding on the 20% vermicompost treatment, and it was not statistically evaluated. Among the other feeding parameters, the duration of Np, D, and E1 did not differ among treatments (Table 2). 

Furthermore, not all the tested *D. citri* had sustained E2 (but all insects have other variables). For the tested 12 individuals for each treatment, 12, 12, 8, and 9 individuals showed sustained E2 for the control, 20%, 40%, and 60% vermicompost rate, respectively.

For other investigated variables, the minimum duration of C before first E1, the time to first E1, the time to first E2, and the average duration of E2 were affected by the vermicompost amendment rate. These four variables, however, were unaffected by the lower rates and only increased by a 60% amendment rate. There was no effect of vermicompost treatments on the first time to the first probe. (Table 3).

### 3.2. Activities of SA and JA Pathway-Related Enzymes

**Figure 1:**
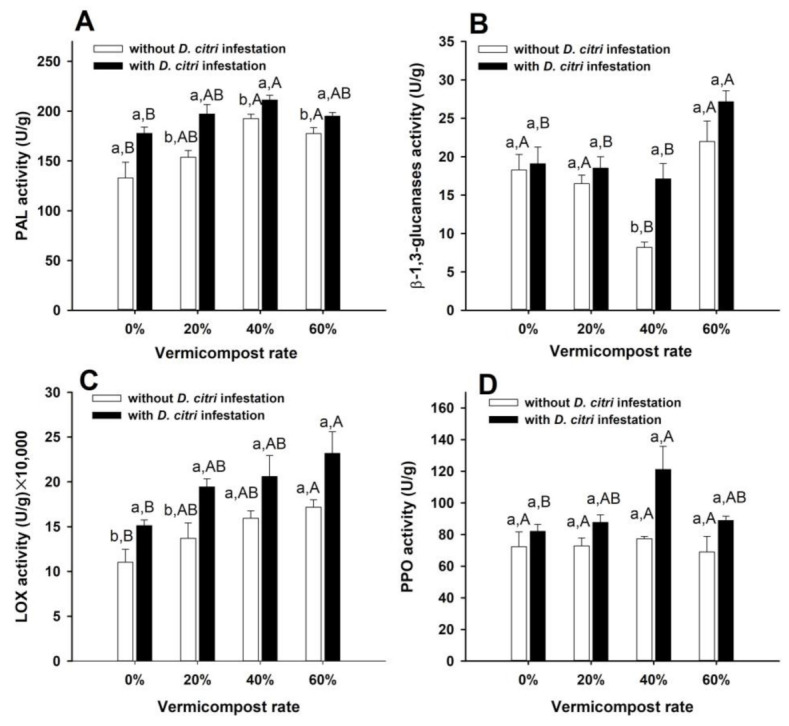
Activities of PAL (**A**), β-1,3-glucanases (**B**), LOX (**C**), and PPO (**D**) in *C. limon* as affected by different rates of vermicompost amendment and *D. citri* infestation. Values are indicated as mean ± SE (*n* = 4). Within the same vermicompost amendment rate, different lowercase letters show significant difference between *D. citri* uninfested and infested plants. With the same infestation state, the different capital letters indicate the differences among the vermicompost treatments. When *p* < 0.05, a difference was considered significant.

For plants without *D. citri* infestation, the vermicompost amendment rate had an impact on the activities of PAL (F = 7.71, df = 12, *p* = 0.04), β-1,3-glucanases (F = 10.32, df = 12, *p* = 0.001), and LOX (F = 4.61, df = 12, *p* = 0.02), but not PPO (F = 4.78, df = 12, *p* = 0.88). specifically, the two higher amendment rates (40% and 60%) boosted the activity of PAL. In addition, the 40% amendment rate decreased β-1,3-glucanases, and the 60% amendment rate increased LOX compared with the unamendment control (Figure 1). 

For plants infested with *D. citri*, the vermicompost amendment rate affected the activities of all tested defense enzymes (PAL: F = 4.70, df = 12, *p* = 0.02, β-1,3-glucanases: F = 6.29, df = 12, *p* = 0.01, LOX: F = 3.57, df = 12, *p* = 0.047, PPO: F = 4.78, df = 12, *p* = 0.02). In detail, the 40% amendment rate increased the activity of PAL and PPO, while the 60% amendment rate increased the activity of β-1,3-glucanases and LOX compared with the control (Figure 1). 

When compared to the non-infested plants, *D. citri* infestation affected the enzyme activities of PAL, β-1,3-glucanases, and LOX in plants, but did not affect that of PPO. Specifically, *D. citri* increased the PAL activity at three amendment rates (20%: t = −3.76, df = 6, *p* = 0.01, 40%: t = −2.90, df = 6, *p* = 0.03, 60%: t = −2.50, df = 6, *p* = 0.046), increased β-1,3-glucanases activity at the 40% amendment rate (t = −4.23, df = 6, *p* = 0.01), and increased LOX activity at two amendment rates (0%: t = −2.61, df = 6, *p* = 0.04, 20%: t = −2.96, df = 6, *p* = 0.03). 

However, there was no interaction effect between the vermicompost amendment rate and *D. citri* infestation on the activities of PAL (F = 1.76, df = 3, *p* = 0.18), β-1,3-glucanases (F = 2.00, df = 3, *p* = 0.14), LOX (F = 0.17, df = 3, *p* = 0.92), and PPO (F = 1.91, df = 3, *p* = 0.16) (Figure 1). 

## 4. Discussion

Vermicompost has been highlighted as an environmentally friendly supplement that can suppress pests and disease sustainably [1]. Indeed, various studies have been conducted to assess the effects of vermicompost amendments on pest performance [4,28]. In this study, by investigating how vermicompost affects *D. citri* feeding behavior and plant defensive enzymes, we provide clear evidence of why vermicompost has a negative effect on pest insect performance.

The phloem phase is considered as the predominant activity of *D. citri* feeding behavior, due to their nutritional requirements [29] and ability to transit HLB [17]. Our EPG study showed that amending citrus with vermicompost disrupts the feeding behaviors of *D. citri*, especially those variables associated with the phloem-feeding phase. Particularly at the higher tested rates, a shorter E2 duration was found on plants receiving vermicompost treatments, demonstrating that *D. citri* withdraw their stylet from phloem sap after a very short ingesting period. The reduced phloem-feeding phase may also relate to the longer pathway phase duration of *D. citri* feeding on the vermicompost treated plants. Similarly, a reduced phloem sap ingestion duration was observed on *D. citri* feeding on citrus plants drenched with the insecticides thiamethoxam and imidacloprid [26]. Previously, the duration of phloem sap was linked to the performance of piercing–sucking insects [30,31,32]. Therefore, our findings help to explain the negative effect of vermicompost on piercing–sucking insects from the aspect of feeding behavior. Furthermore, we note that the effect of vermicompost on *D. citri* varied with the amendment rate, suggesting that a relatively high rate of vermicompost may be required to control *D. citri*.

Plant resistance has been linked to an increase in the defense compounds [33], and the defensive enzymes associated with the SA and JA pathways play a key role in plant defense against piercing–sucking insects [34,35]. For example, the overexpression of PAL, a rate-limiting enzyme of the SA pathway, can increase plant SA content and increase the mortality of brown planthopper *Nilaparvata lugens* [36]. The β-1,3-glucanases, a defensive enzyme downstream of the SA pathway, mediates the resistance of wheat against *Diuraphis noxia* [37]. LOX is the rate-limiting enzyme of the JA pathway, and the inhibition of LOX increased the fecundity of *Rhopalosiphum padi* and *M. persicae* [38]. PPO is a defensive enzyme downstream of the JA pathway, and it is shown to mediate the negative effect of MeJA on the population of *M. persicae* [39]. Moreover, SA application can activate PAL and β-1,3-glucanases activity in citrus plants [40]. LOX and PPO were positively correlated with plant JA content [41,42]. Here, the PAL and PPO activity on *D. citri*-infested plants were enhanced by vermicompost (40% amendment rate). Similarly, β-1,3-glucanases and LOX activity were enhanced at the 60% vermicompost amendment rate compared with the control. This indicate that these two rates of vermicompost enhanced the SA and JA pathway-related enzyme activities. Previously, we showed that the SA and JA-related defense reduced the duration of E2 and made it more difficult for *D. citri* to acquire phloem sap [13]. The phenomenon that SA and JA-related defenses suppress feeding has also been investigated in other piercing–sucking insects such as the aphid [43] and whitefly [44]. Therefore, it is speculated that the disruption in *D. citri*’s feeding behavior resulting from the addition of vermicompost may be caused by the increased activity of enzymes involved in the SA/JA pathway.

The feeding behavior of piercing–sucking insects is suggested to be tightly related to the transmission of insect-vectored pathogens, which have been studied in insects including the aphid [45], whitefly [46], and psyllid [47]. Accordingly, because *C*Las is present only in the phloem, for example, *D. citri* inoculates bacteria into the plant by salivating into the phloem (waveform E1) and acquires bacteria during the ingestion of phloem sap (waveform E2) [47]. Particularly, E2 more than 10 min was used to estimate the possibility to acquire *C*Las [48]. Here, neither the number nor the duration of E1 were significantly altered, but vermicompost did reduce the number and duration of E2/sustained E2. This suggests that vermicompost may limit *D. citri*’s ability to acquire CLas. Additionally, some *D. citri* at the 40% and 60% amendment rates failed to exhibit any consistent E2, making the acquisition of CLas possibly implausible. Vermicompost therefore has a significant potential to reduce *D. citri*’s acquisition of CLas, as demonstrated by the interference of the EPG variables, and it should be further researched in the field and in the *D. citri*-CLas-citrus system.

## 5. Conclusions

This study discovered that adding vermicompost can reduce *D. citri*’s ability to feed on citrus trees, which may be because of increased SA and JA pathway-related enzyme activity. The results of this study support the use of defense enzymes and feeding behavior as key indicators for assessing the management impact of vermicompost on *D. citri*. Furthermore, this study supports the use of vermicompost for the organic management of both *D. citri* and HLB.

## Figures and Tables

**Table 1:** Number of waveform event per insect (NWEI) of *D. citri* under vermicompost amendment.

EPG Variables	Control	20% Vermicompost	40% Vermicompost	60% Vermicompost	F	df	*p*
Np	1.25 ± 0.18 a	1.33 ± 0.26 a	1.33 ± 0.33 a	1.17 ± 0.11 a	0.12	44	0.95
C	6.20 ± 1.63 a	3.33 ± 0.65 a	3.33 ± 0.50 a	3.25 ± 0.79 a	2.20	44	0.10
D	5.25 ± 1.32 a	2.50 ± 0.57 a	2.58 ± 0.53 a	2.500 ± 0.73 a	2.58	44	0.07
E1	5.25 ± 1.32 a	2.50 ± 0.57 a	2.58 ± 0.53 a	2.500 ± 0.73 a	2.58	44	0.07
E2	2.8 ± 0.55 a	1.75 ± 0.43 a	1.83 ± 0.51 a	1.50 ± 0.20 a	1.53	44	0.22
E2 > 10 min	1.67 ± 0.28 a	1.33 ± 0.19 ab	0.83 ± 0.21 b	0.75 ± 0.13 b	4.25	44	0.01
G	0 a	0.17 ± 0.112 a	0 a	0 a	2.20	44	0.10

Values are indicated as mean ± SE (*n* = 12). Different lowercase letters in the same row indicate the significant differences among the treatments (*p* < 0.05).

**Table 2:** Waveform duration per insect (WDI, min) of *D. citri* under vermicompost amendment.

EPG Variables (min)	Control	20% Vermicompost	40% Vermicompost	60% Vermicompost	F	df	*p*
Np	17.78 ± 10.92	4.86 ± 1.899 a	2.60 ± 0.83 a	1.92 ± 0.75 a	1.78	44	0.17
C	314.31 ± 34.41 b	349.08 ± 23.80 b	432.44 ± 9.70 a	451.37 ± 4.26 a	9.26	44	<0.001
D	4.57 ± 1.32 a	2.76 ± 1.01 a	2.43 ± 0.61 a	2.66 ± 0.66 a	1.09	44	0.36
E1	5.23 ± 1.26 a	5.80 ± 2.43 a	2.21 ± 0.53 a	3.25 ± 1.31 a	1.19	44	0.33
E2	138.12 ± 29.17 a	116.11 ± 20.46 a	40.32 ± 9.75 b	20.41 ± 3.63 b	9.48	44	<0.001
E2 > 10 min	133.53 ± 30.21 a	115.11 ± 20.46 a	52.44 ± 9.83 ab	24.31 ± 3.24 b	5.53	37	0.003
G	NA	8.43 ± 0.55	NA	NA	-	-	-

Values are indicated as mean ± SE (*n* = 8 at 40% vermicompost and *n* = 9 at 60% vermicompost for E2 > 10 min, *n* = 12 for other values). Different lowercase letters in the same row indicate the significant differences among the treatments (*p* < 0.05).

**Table 3:** Other feeding behavior variables associated with plant resistance to *D. citri* under vermicompost amendment.

EPG Variables (min)	Control	20% Vermicompost	40% Vermicompost	60% Vermicompost	F	df	*p*
Time to first probe from start of recording	10. 50 ± 5.47 a	14.86 ± 10.98 a	2.10 ± 0.76 a	1.66 ± 0.71 a	1.11	44	0.36
Minimum duration of C before first E1	112.34 ± 21.58 b	135.77 ± 32.21 ab	150.60 ± 26.50 ab	257.61 ± 40.18 a	4.33	44	0.01
First E1 from start of recording	130.75 ± 21.76 b	152.87 ± 31.13 ab	151.33 ± 26.49 ab	259.11 ± 40.33 a	3.56	44	0.02
First E2 from start of recording	138.36 ± 23.30 b	154.12 ± 31.11 ab	171.66 ± 31.73 ab	270.01 ± 38.22 a	3.53	44	0.02
Average duration of E2	84.17 ± 29.07 b	82.46 ± 17.08 ab	30.46 ± 9.46 ab	18.83 ± 4.04 a	3.92	44	0.01

Values are indicated as mean ± SE (*n* = 12). Different lowercase letters in the same row indicate the significant differences among the treatments (*p* < 0.05).

## Data Availability

The data presented in this study are available upon request from the corresponding author.
